# Alendronate as an Effective Treatment for Bone Loss and Vascular Calcification in Kidney Transplant Recipients

**DOI:** 10.1155/2014/269613

**Published:** 2014-02-19

**Authors:** Masanori Okamoto, Shintaro Yamanaka, Wataru Yoshimoto, Takashi Shigematsu

**Affiliations:** Division of Nephrology, Department of Internal Medicine, Wakayama Medical University, 811-1 Kimiidera, Wakayama City, Wakayama 641-0012, Japan

## Abstract

Kidney transplant recipients develop secondary osteoporosis induced by immunosuppressive medication, with a high risk of fracture, and abdominal aortic calcification (AC) is a known predictor of cardiovascular mortality. In this study of 12 stable kidney recipients, we estimated the preventive effect of bisphosphonate treatment on bone loss and progression of AC. We randomly divided the subjects into a treatment group with alendronate (group A: 5 subjects) and a control group (group C: 7 subjects). Group A patients received 35 mg/week of alendronate over 24 months, while group C patients were not administered with any bisphosphonates. Two major endpoints were established: (1) the time-dependent change in bone mineral density (BMD) estimated with DEXA and (2) progression of abdominal AC, calculated twice as an index (ACI) using computed tomography data. Over the 2-year study period, group A patients showed significantly increased BMD of 1.86 ± 0.85% (*P* = 0.015 versus baseline), and almost complete inhibition of ACI progression (38.2 ± 24.2% to 39.6 ± 24.3%), but group C patients showed a decrease in BMD decline with bone loss and progression of ACI (32.8 ± 25.0% to 37.8 ± 29.2%, *P* = 0.061). In conclusion, alendronate therapy was an effective treatment in kidney transplant recipients for secondary osteoporosis and vascular calcification as ectopic calcification. This clinical trial is registered with number JMA-IIA00155 of JMACCT CTR.

## 1. Introduction

Renal transplantation is the gold standard therapy for end-stage renal failure, but the subsequent bone loss is related to adverse effects of immunosuppressive drugs on bone remodeling and bone quality [1, 2]. Osteoporosis and fragility fractures are serious complications of renal transplantation [3], and the choice of immunosuppressive drug, malnutrition, adynamic bone disease, and secondary hyperparathyroidism are important factors in the development of posttransplant osteoporosis [4]. Mineral and bone disorders following kidney transplantation are common and characterized by loss of bone volume and mineralization abnormalities, often leading to low turnover bone disease [5]. The incidence of fractures in kidney transplant recipients is threefold higher than in dialysis patients [6–8]. Fracture rates and bone mineral density (BMD) are associated with secondary osteoporosis, although not as strongly as with primary osteoporosis. Glucocorticoids are potent skeletal toxins, with even small doses causing a significant decrease in bone mass and increased fracture rate in previously healthy bone [9], and calcineurin inhibitors are also linked to osteoporosis [10, 11]. Bisphosphonates can prevent bone loss and fractures in patients with postmenopausal osteoporosis [12] or glucocorticoid-associated osteoporosis [13].

Accumulating epidemiologic evidence suggests that osteoporosis coexists with cardiovascular disease [14–16]. Progression of aortic calcifications associated with faster bone loss [16, 17] and low BMD has been shown to predict cardiovascular events and cardiovascular mortality [18, 19]. In chronic kidney disease (CKD), observational studies have shown a strong inverse correlation between BMD and cardiovascular mortality, as well as vascular calcification [20, 21]. Moreover, calcium apatite, which forms the crystal component of bone, has been found to accumulate in calcified blood vessels of many patients with CKD, and calcified plaques also express several bone matrix proteins that stimulate vascular smooth muscle cells to differentiate into osteoblasts. These findings support an important interaction between bone disorders and calcification of soft tissues [22, 23].

In the early posttransplantation period, observational studies have shown progression of both coronary and aortic vascular calcifications in kidney recipients [24, 25], a finding that possibly is linked to accelerated bone loss over the same period. Because several studies have shown that bisphosphonates can directly inhibit medial vascular calcification independent of bone resorption [26–28], we examined the preventive effect of bisphosphonate therapy on bone loss and progression of aortic calcification in kidney transplant recipients.

## 2. Materials and Methods

We enrolled 12 patients (8 men and 4 women) with stable allograft function (defined by serum creatinine <2.0 mg/dL) for at least 1 year and conducted a prospective study. We randomly divided the subjects into two groups: a treatment group with alendronate 35 mg/week for 24 months (group A: 5 subjects) and a control group (group C: 7 subjects) not given any bisphosphonates.

Two major endpoints were established: (1) time-dependent change in BMD, estimated from dual-energy X-ray absorptiometry (DEXA) scans of the whole body, and (2) progression of abdominal aortic calcification assessed by twice calculating an index (ACI) using computed tomography data. All patients underwent measurement of serum calcium, phosphate, bone-specific alkaline phosphatase (BAP), vitamin D metabolites, N-terminal telopeptides of bone-specific type I collagen (NTx), and whole parathyroid hormone (wPTH) levels at baseline and at 12 and 24 months. Allograft function was evaluated by measuring estimated glomerular filtration rate (eGFR) based on serum creatinine.

### 2.1. Statistical Analysis

All analyses and calculations were performed with SPSS software, version 20 (SPSS, Chicago, IL, USA). All values are expressed as the mean ± standard error of the mean (SEM). Statistical analyses were performed using ANOVA and subsequent Tukey's simultaneous multiple comparison test. Differences at *P* < 0.05 were considered statistically significant.

## 3. Results

**Figure 1:**
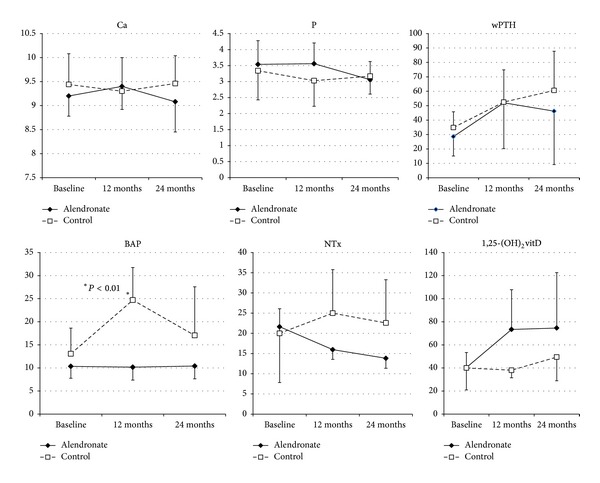
Baseline and follow-up values of parameters in bone turnover markers.

**Figure 2:**
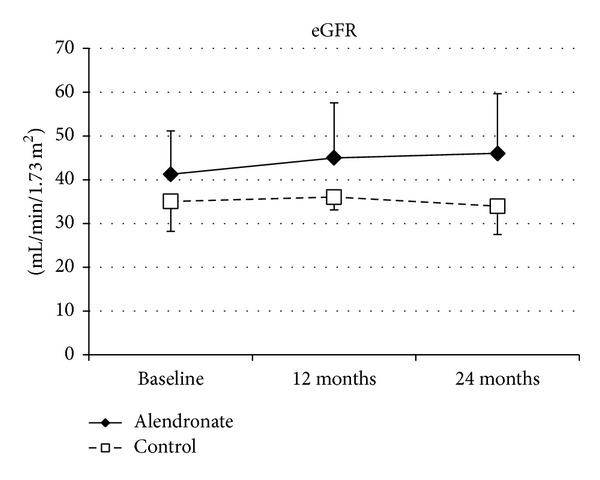
Baseline and follow-up values of parameters in allograft function. eGFR: estimated glomerular function.

**Figure 3:**
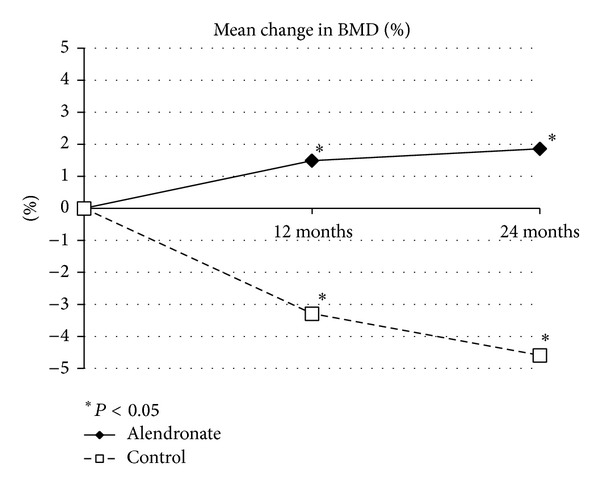
Mean change in bone mineral density (BMD).

**Figure 4:**
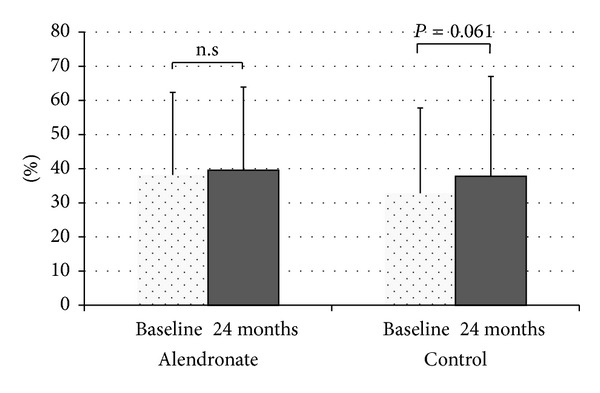
Change in aortic calcification index.

Patients in group A did not experience any adverse events during the study period. There were no differences between groups in the clinical characteristics of the patients (Table 1). There were no significant differences between groups in baseline serum calcium, phosphate, BAP, wPTH, 1,25-dihydroxycholecalciferol, and NTx levels, or eGFR (Table 2). There were also no significant temporal changes in serum calcium and phosphate levels, or eGFR, in either group immediately after the treatment period (Figures 1 and 2). Mean wPTH levels in group C showed a gradual increase during the study, but there were no significant between-group differences after the treatment period (Figure 1). Mean BAP levels in group C tend to be temporarily increased by 12 months, but decreased by 24 months, and there were no significant between-group differences after the treatment period. Serum 1,25-dihydroxycholecalciferol and NTx levels did not differ significantly between the treatment and control groups at 24 months (Figure 1). Over the 2-year study period, group A patients showed significantly increased BMD of 1.86 ± 0.85% (*P* = 0.015 versus baseline, Figure 3) and almost completely inhibition of progression in the ACI (38.2 ± 24.2% to 39.6 ± 24.3%, Figure 4). By contrast, group C patients showed a decline in BMD with bone loss (Figure 3) and progression of the ACI (32.8 ± 25.0% to 37.8 ± 29.2%, *P* = 0.061, Figure 4).

## 4. Discussion

Osteoporosis is a frequent complication of renal transplantation. Osteoporotic fractures reduce quality of life, increase morbidity and mortality, and increase healthcare costs [29]. The risk of the development and progression of vascular calcification after renal transplantation is well described in the literature. In this preliminary study, in which we compared the effect of alendronate therapy versus no bisphosphonates on BMD loss after kidney transplantation, we found significant protection of BMD in the treatment group after 12 and 24 months. At the same time, alendronate therapy showed a tendency to inhibit the progression of aortic calcification in kidney transplant recipients.

Renal transplantation is now a reasonably common and successful procedure. As the number of transplant recipients has grown, new challenges have arisen in the management of long-term complications of transplantation. Posttransplantation bone disease is a such important complication, because it occurs in a substantial proportion of patients. As a consequence, bone mass loss and bone fractures are common in kidney transplant patients and cause substantial morbidity. Among kidney recipients, the most important cause of osteoporosis is corticosteroid treatment.

Bisphosphonates inhibit resorption of bone by binding preferentially to skeletal sites where turnover rates are high, such as trabecular bone, and by directly suppressing the number and activity of osteoclasts. Also, they prevent the apoptosis of osteoblasts and osteocytes induced by glucocorticoids [30]. Bisphosphonates (risedronate, alendronate, ibandronate, zoledronic acid, and pamidronate) have been shown in numerous studies to prevent BMD loss after renal transplantation [31–37], but generally there has not been evidence of a reduction in the rate of fracture [32, 38]. The latter may reflect a lack of statistical power in the previous studies or may result from a true absence of an effect of bisphosphonates on improving bone strength after transplantation, despite attenuating BMD losses [39]. A significant concern with regard to bisphosphonate use after transplantation is the potential to prolong or induce adynamic bone. Few studies have included bone biopsy data, but in the study by Coco et al. [32], which demonstrated that pamidronate use was associated with preservation of BMD at both the femoral neck and lumbar spine, there was an increase in biopsy-proven adynamic bone disease after 6 months compared with placebo. It is unknown whether the increase in adynamic bone was detrimental or if the increase in BMD decreased the fracture risk. In the present study, we showed that alendronate therapy increased the BMD of the whole body in kidney transplant recipients.

Cardiovascular disease remains the leading cause of mortality in both patients on dialysis and those with a functioning renal transplant [40, 41]. Vascular calcification independently predicts cardiovascular disease, which is the major cause of death in kidney transplant recipients. According to animal studies, bisphosphonates generally inhibit vascular calcification in a variety of models [42]. The first report of the effects of bisphosphonates on vascular calcification was in the 1970s, with experiments showing inhibition of soft tissue calcification in both animals and humans [43, 44]. Those data have been confirmed by more recent animal studies using several bisphosphonates at varying doses [45–47].

The exact mechanism by which bisphosphonates inhibit vascular calcification is unclear. It may be by inhibition of bone resorption, with the reduced efflux of calcium and phosphate limiting their availability for deposition in the vasculature [48]. A new study has shown that the osteoblastic differentiation of rat aortic vascular smooth muscle cells (VSMCs) is significantly reduced by the action of alendronate in a dose-dependent manner [49]. Another research indicates that alendronate inhibits artery calcification by upregulating osteopontin and osteoprotegerin expression in rat model [50]. Alternatively, bisphosphonates may have direct effects on the vessel wall and, similar to pyrophosphate, on crystal formation. There have been varying responses in clinical studies; studies performed in the general population have reported no difference in vascular calcification with bisphosphonate administration; however, according to the scarce clinical data from patients with CKD, these drugs can improve vascular calcification [27, 28, 51].

Bisphosphonate are potent inhibitors of bone turnover [39, 52] and, at least in CKD patients, low bone turnover (i.e., adynamic bone disease) is associated with vascular calcification [31]. Previous work revealed a complex association between bisphosphonate use and cardiovascular calcification. According to a recent study, bisphosphonate use was associated with a high prevalence of cardiovascular calcification in woman aged <65 years [53].

Despite some data supporting a role for bisphosphonates in the management of vascular calcification, additional clinical studies of their use in kidney transplant recipients are required. In this study, we evaluated the preventive effect of bisphosphonate on bone loss and progression of aortic calcification. Although there is no well-established therapeutic approach to the management of bone and mineral disorders in renal transplant recipients, clinicians should continuously individualize therapy for their patients.

## 5. Conclusions

The present study demonstrated that the alendronate therapy is a desirable treatment for secondary osteoporosis with vascular calcification as ectopic calcification in kidney transplant recipients. However, the effect of bisphosphonates on fracture risk and patient mortality is still obscure and requires further large-scale study.

## Figures and Tables

**Table 1:** Baseline characteristics of patients.

	Alendronate (*n* = 5)	Control (*n* = 7)	*P* value
Age (years)	52.8 ± 12.6	52.9 ± 7.3	NS
Male (%)	80	57	NS
Diabetes (%)	40	43	NS
HTN (%)	80	71	NS
Tac (%)	80	86	NS
Steroid (mg/day)	5	4.7	NS
HD vintage (months)	136.8 ± 142.3	71.1 ± 76.6	NS
Transplant vintage (months)	59.6 ± 58.5	45.3 ± 42.3	NS

HTN: hypertension; Tac: tacrolimus; NS: not significant.

**Table 2:** Biochemical parameters and bone turnover markers at baseline.

	Alendronate (*n* = 5)	Control (*n* = 7)	*P* value
Ca (mg/dL)	9.2 ± 0.4	9.4 ± 0.5	NS
P (mg/dL)	3.5 ± 0.6	3.3 ± 0.8	NS
wPTH (pg/mL)	28.5 ± 9.7	34.8 ± 12.4	NS
BAP (IU/L)	10.3 ± 2.3	13.0 ± 5.1	NS
1, 25-(OH)_2_vitD	40.3 ± 11.6	40.0 ± 17.6	NS
NTx	21.6 ± 12.3	20.0 ± 5.6	NS
eGFR (mL/min/1.73 m^2^)	41.2 ± 9.9	35.1 ± 6.9	NS

Ca: serum calcium; P: serum phosphate; wPTH: serum whole parathyroid hormone; BAP: serum bone-specific alkaline phosphatase; 1, 25-(OH)_2_vitD: serum 1, 25-dihydroxycholecalciferol; NTx: serum N-terminal telopeptides of bone-specific type I collagen; eGFR: estimated glomerular filtration rate; NS: not significant.
